# Independent and combined associations of maternal and own smoking with adult lung function and COPD

**DOI:** 10.1093/ije/dyy221

**Published:** 2018-10-17

**Authors:** Maria C Magnus, John Henderson, Kate Tilling, Laura D Howe, Abigail Fraser

**Affiliations:** 1 MRC Integrative Epidemiology Unit at the University of Bristol, Bristol, UK; 2 Department of Population Health Sciences, Bristol Medical School, Bristol, UK; 3 Centre for Fertility and Health, Norwegian Institute of Public Health, Oslo, Norway; 4 NIHR Bristol Biomedical Research Centre at the University Hospitals Bristol NHS Foundation Trust and the University of Bristol, Bristol, UK

**Keywords:** chronic obstructive pulmonary disease, spirometry, maternal, smoking, UK Biobank

## Abstract

**Background:**

Limited evidence suggests that exposure to maternal smoking *in utero* or early life might be associated with chronic obstructive pulmonary disease (COPD), but whether this is independent of later own smoking remains unclear. Our objective was to examine the independent and combined association of maternal and own smoking with adult lung function and COPD.

**Methods:**

We used UK Biobank to examine associations of maternal smoking around delivery, and pack-years of own smoking, with lung function (*n* = 502 626) and hospitalization/death from COPD (*n* = 433 863). We calculated the additive interaction between maternal and own smoking on the outcomes of interest, and estimated the association with maternal smoking within categories of own smoking.

**Results:**

There was no strong evidence that maternal smoking influenced adult lung health among never smokers. Exposure to both maternal and own smoking was associated with lower Forced expiratory volume (FEV_1_)/ forced vital capacity (FVC) and greater risk of hospitalization/death from COPD than expected from their independent associations. For FEV_1_/FVC, the mean difference according to maternal smoking was –0.02 (–0.06, 0.02), –0.01 (–0.05, 0.03), –0.11 (–0.16, –0.05) and –0.11 (–0.19, –0.04) among women who smoked ≤10, 11–20, 21–30 and >30 pack-years, respectively. The association between maternal smoking and COPD also varied by pack-years of own smoking, with a hazard ratio of 2.25 (1.30, 3.89) for ≤10 years, 1.23 (0.80, 1.89) for 11–20 years, 1.30 (0.85, 2.01) for 21–30 years and 1.14 (0.91, 1.43) for >30 years.

**Conclusions:**

Our findings indicate an excess reduction in FEV_1_/FVC and risk of COPD due to maternal smoking that is heterogeneous across levels of own smoking.

**Key Messages**

- Our findings from UK Biobank show that exposure to both maternal and own smoking resulted in a reduction in lung function and increased risk of chronic obstructive pulmonary disease (COPD) that exceeded what was expected based on their independent associations.
- The magnitude of the association between maternal smoking and forced expiratory volume (FEV_1_)/forced vital capacity (FVC) increased with greater number of pack-years of own smoking, whereas the magnitude of the association between maternal smoking and hospitalization/death from COPD was greatest among those who had smoked <10 pack-years.
- These results suggest that public-health initiatives supporting the current trend of reduction in smoking may have benefits for the lung health of two generations.

## Introduction

The main modifiable risk factor for chronic obstructive pulmonary disease (COPD) is own smoking. However, a sizable proportion (25–45%) of COPD occurs among never smokers.^1^ It is therefore important to identify further contributing causes of COPD. Based on the observation that environmental exposures during pregnancy and early childhood influence lung function in early life,^2–4^ which shows a strong degree of tracking throughout the life course,^5^ it is plausible that early-life environment affects COPD risk.

Maternal smoking during pregnancy is associated with increased wheezing illness and diminished lung function during childhood.^6^ It remains uncertain whether maternal smoking might also have a continued impact on adult lung health. A few previous studies stemming from two independent cohorts looked at maternal smoking and adult lung function.^7–9^ These studies indicate an association between maternal smoking and adult lung function that was seemingly independent of own smoking after traditional multivariable adjustment.^7–9^

Two of the previous studies suggest that the combined exposure to maternal and own smoking resulted in an excess reduction in lung function/greater rate of lung-function decline.^7^^,^^9^ Only one study examined maternal smoking in relation to COPD, but this study could not examine independent and combined associations with maternal and own smoking with COPD due to its limited sample size.^7^ It therefore remains unclear whether there might be an interaction between these two exposures on COPD risk, and what the direct effect of maternal smoking on COPD risk might be in the presence of an interaction.

The objective of the current study was to examine the independent and combined associations of maternal and own smoking with adult lung function and hospitalization/death from COPD in the UK Biobank cohort.

## Methods

### Study population

We studied participants in UK Biobank, including 503 325 people between 40 and 69 years of age, who were recruited between 2006 and 2010, from 22 assessment centres across England, Scotland and Wales.^10^^,^^11^ The participation rate was 5% and all participants gave written informed consent. Participants were followed through national hospital and death registers. Information from both registries was available until 28 February 2015 for England, 16 March 2015 for Wales and 28 October 2014 for Scotland. Ethical approval for UK Biobank was obtained from the NHS National Research Ethics Service (Ref 11/NW/0382). The data available included 502 629 individuals due to withdrawals. After excluding 3 individuals registered as deceased before enrolment, 502 626 were eligible for the current study.

### Exposure

Participants were asked: Did your mother smoke regularly around the time when you were born? The answer options were ‘no’, ‘yes’ and ‘don’t know’. Based on self-reported information on age at smoking initiation, average number of cigarettes/cigars/pipes smoked per day, age of smoking cessation (for former smokers) and age at recruitment (for current smokers), we calculated the number of pack-years of smoking at baseline. Pack-years of own smoking was categorized as none, up to 10 years, between 11 and 20 years, between 21 and 30 years, and more than 30 years. The exposure consisted of 10 mutually exclusive categories of maternal and pack-years of own smoking.

### Spirometry measurements

Forced expiratory volume (FEV_1_) and forced vital capacity (FVC) were measured at baseline using Vitalograph Pneumotrac 6800 (Vitalograph, UK). Spirometry was not conducted if participants had experienced a chest infection in the last month; had a life-time history of detached retina or collapsed lung; if they had been through a heart attack, eye surgery or surgery to chest or abdomen in last 3 months; or if they were currently pregnant or on tuberculosis medications. If the reproducibility of the first two measurements was adequate, defined as a ≤ 5% difference in FVC and FEV_1_, a third measurement was not required. Post-bronchodilator spirometry was not available, although drug treatment was not withheld. The spirometry measurements were internally standardized by age, sex and height before analysis.

### COPD

COPD at baseline was defined based on self-report, historical diagnosis in the national hospital registers and/or a FEV_1_/FVC <0.7 according to the Global Initiative for Chronic Obstructive Lung Disease (GOLD) criteria.^12^ Incident hospitalization/death from COPD after baseline was identified from national registers. The registers are coded according to the International Classification of Diseases (ICD). ICD codes that were used to define COPD included ICD-9 codes 490–492, 494 and 496, in addition to ICD-10 codes J40–44.

### Covariates

**Figure 1.**
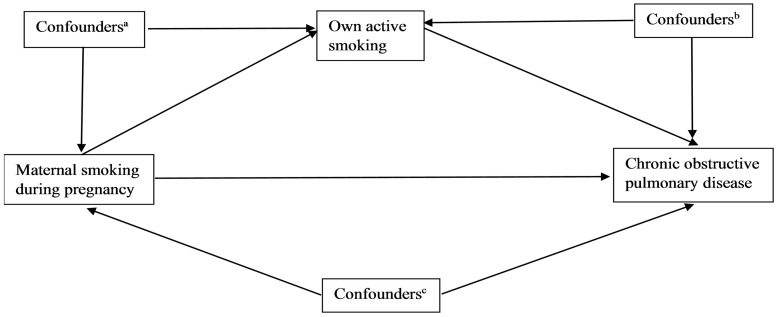
Simplified illustration of the underlying theoretical framework. ^a^Potential confounders of the association between the exposure and the mediator. Participants’ own socio-economic status and ethnicity were used as a proxies for maternal socio-economic status and ethnicity. ^b^Potential confounders of the association between the mediator and outcome. Available measures included age, sex, ethnicity, socio-economic status, BMI and history of asthma. ^c^Confounders of the association between the exposure and outcome. Participants’ own socio-economic status and ethnicity were used as proxies for maternal socio-economic status and ethnicity. Available measures for socio-economic status included qualifications, income and Townsend area-level deprivation index.

Additional information obtained by self-report included age, sex, ethnicity (European vs other), educational qualifications (college, university or other professional degree, Advance levels/Advance Subsidiary levels or equivalent, Ordinary levels/General Certificate of Secondary Educations or equivalent, National Vocational Qualifications, Higher National Diploma, Higher National Certificate or equivalent, other), average household income (less than £18 000, £18 000–30 999, £31 000–51 999, £52 000–100 000, >£100 0000 and ‘prefer not to answer/don’t know’) and Townsend area-level deprivation index. Nurses measured participants’ height and weight at baseline, which was subsequently used to calculate body mass index (BMI; weight in kilograms/height in meters^2^). Asthma at baseline was defined using self-report and historical diagnosis in the national hospital registers (ICD9 code 493; ICD10 codes JJ45 and JJ46). A simplified illustration of the underlying theoretical framework is provided in Figure 1. Notably, it is not possible to illustrate a potential interaction between maternal and own smoking on offspring adult lung health in such a theoretical framework.

### Statistical analysis

We imputed 20 datasets with missing covariate information using multiple imputation by fully conditional specification (chained equations).^13^ The amount of missing information on individual covariates ranged from none (age, sex and asthma at baseline) to 28% (spirometry measurements). For individuals who responded ‘don’t know’ to whether their mother smoked (14%), we set the value to missing and subsequently imputed the value for maternal smoking. The imputation model included the covariates described above, birthweight, parental history of COPD, country of residence and interaction terms for maternal and own smoking. We present the results from the complete-case analysis in the supplement, and the study population available for this analysis is illustrated in Supplementary Figure 1, available as Supplementary data at *IJE* online.

We examined maternal and pack-years of own smoking in relation to the standardized spirometry measurements using linear regression, reporting mean differences (β) and 95% confidence intervals (CIs). We calculated the associations of the mutually exclusive exposure categories of maternal and pack-years of own smoking with incident hospitalization/death from COPD using Cox proportional hazards regression, reporting hazard ratios (HRs) and 95% CI. We also tested for heterogeneity in the associations of maternal and own smoking with adult lung health by sex.

Instead of conventional multivariable adjustment for potential confounders, we used marginal structural models (MSMs), since some of the confounders of the association between own smoking and adult lung health could be influenced by maternal smoking (such as asthma and BMI).^14–16^ The inverse probability weight for maternal smoking was calculated from a logistic regression model including the participant’s age, sex, ethnicity, qualifications, income and Townsend area-level deprivation index. The inverse probability of pack-years of own smoking was calculated from a multinomial logistic regression model adjusting for these same background characteristics, in addition to maternal smoking, BMI and asthma at baseline.^14–16^ We subsequently stabilized these two weights before using their product as the final analytical weight.

We were interested in an additive interaction between maternal and own smoking, which was estimated by including product terms in the linear regression for the spirometry measurements, whereas the relative excess risk due to interaction (RERI) was calculated as a measure of additive interaction for hospitalization/death from COPD.^17^ Since we found some evidence of an interaction, we then estimated the association with maternal smoking within levels of own smoking, by adding together the coefficients for maternal smoking and the interaction terms for each group of own smoking, and calculating the CIs using the delta method.^16^

As a secondary analysis, we estimated the associations of maternal and own smoking with COPD at baseline using logistic regression, reporting odds ratios (ORs) and 95% CIs. Since the spirometry measurements were not done post bronchodilation, and we therefore did not know whether the airflow obstruction was reversible or not, we also conducted a sensitivity analysis excluding individuals with asthma at baseline from the analysis of prevalent and incident COPD. All analyses were conducted in Stata version 14 (Statacorp, Texas).

## Results

The distribution of background characteristics according to maternal smoking in the imputed dataset is shown in Table 1. Participants who reported that their mother smoked around the time of their delivery were more likely to be male, to be of European ethnicity, to have lower educational qualifications, to have lower income, to have smoked themselves, to have a higher BMI and to have a history of asthma at enrolment.

**Table 1.** Distribution of background characteristics by maternal smoking around the time of delivery (*n* = 502 626)

Background characteristic	No (*N* = 354 529)	Yes (*n* = 148 097)	*p*-value
**Age (median, IQR)**	58 (50, 64)	57 (50, 62)	<0.001
**Sex (%)**			<0.001
Female	195 005 (55.0)	78 451 (53.0)	
Male	159 524 (45.0)	69 646 (47.0)	
**Ethnicity (%)**			<0.001
European	329 126 (92.8)	146 084 (98.6)	
Other	25 403 (7.2)	2013 (1.4)	
**Qualifications (%)**			<0.001
College, university or other professional	142 429 (40.2)	47 663 (32.2)	
A-levels/AS-levels or equivalent	40 457 (11.4)	15 778 (10.7)	
O-levels/GCSEs or equivalent	74 621 (21.0)	32 618 (22.0)	
CSEs or equivalent	17 434 (4.9)	10 024 (6.8)	
NVQ, HND, HNC or equivalent	21 858 (6.2)	11 626 (7.9)	
Other	57 729 (16.3)	30 387 (20.5)	
**Average household yearly income, pounds (%)**			<0.001
Less than 18 000	68 508 (19.3)	30 418 (20.5)	
18 000–30 999	77 043 (21.7)	32 435 (21.9)	
31 000–51 999	77 646 (21.9)	34 280 (23.1)	
52 000–100 000	61 600 (17.4)	25 453 (17.2)	
>100 000	16 770 (4.7)	6337 (4.3)	
Prefer not to answer/don’t know	52 962 (14.9)	19 175 (12.9)	
**Townsend area-level deprivation index (mean, SD)**	–2.21 (–3.68, 0.42)	–1.96 (–3.53, 0.87)	<0.001
**Pack-years of own smoking (%)**			<0.001
None	234 529 (66.2)	88 517 (59.8)	
Up to 10	33 098 (9.3)	12 600 (8.5)	
Between 11 and 20	33 402 (9.4)	15 086 (10.2)	
Between 21 and 30	23 024 (6.5)	12 484 (8.4)	
More than 30	30 477 (8.6)	19 411 (13.1)	
**Asthma at baseline (%)**			<0.001
No	313 160 (88.3)	128 587 (86.8)	
Yes	41 369 (11.7)	19 510 (13.2)	
**BMI, kg/m^2^ (median, IQR)**	26.5 (24.0, 29.6)	27.2 (24.5, 30.6)	<0.001
**Height, cm (median, IQR)**	168 (162, 175)	168 (161, 175)	<0.001
**FEV1 (median, IQR)**	0.003 (–0.629, 0.623)	0.007 (–0.603, 0.621)	<0.001
**FVC (median, IQR)**	0.037 (–0.601, 0.642)	0.025 (–0.602, 0.624)	<0.001
**FEV1/ FVC (median, IQR)**	0.141 (–0.484, 0.671)	0.092 (–0.556, 0.631)	<0.001
**COPD at baseline (%)**			<0.001
No	307 187 (86.6)	126 677 (85.5)	
Yes	47 342 (13.4)	21 421 (14.5)	
**COPD hospitalization/death (%)**			<0.001
No	306 111 (99.6)	125 974 (99.4)	
Yes	1, 076 (0.4)	703 (0.6)	
Number of years of follow-up from the registries (median, IQR)	6.1 (5.4, 6.7)	6.1 (5.5, 6.7)	<0.001

The distribution of the covariates constitutes an average across the 20 imputed datasets.

The combined exposure to maternal and own smoking was associated with a reduction in both FEV_1_ and FVC that exceeded their independent associations (Table 2). The excess reduction in standardized FEV_1_ was 0.0005, –0.04, –0.08 and –0.09 for <10, 11–20, 21–30 and more than 30 pack-years of own smoking, respectively (Table 2). Exposure to maternal smoking showed some evidence of a positive association with both FEV_1_ and FVC among never smokers, but the mean difference was arguably very modest (Table 3). In contrast, maternal smoking was associated with lower FEV_1_ among individuals who had smoked more than 20 pack-years, with a mean difference of –0.07 (95% CI: –0.12, –0.03) among those who had smoked more than 30 pack-years (Table 3). The results were similar in the complete-case analysis (Supplementary Tables 1 and 2, available as Supplementary data at *IJE* online).

**Table 2.** Multiple-imputation analysis of maternal and pack-years of own smoking with FVC and FEV_1_ at baseline (*n* = 502 626)

Maternal smoking	Pack-years of own smoking	FVC			FEV_1_		
		Median (IQR)	Mean difference (95% CI)^a^	Additive interaction	Median (IQR)	Mean difference (95% CI)^a^	Additive interaction
				Relative excess change (95% CI)			Relative excess change (95% CI)
No	No	0.02 (–0.61, 0.64)	Ref		0.08 (–0.55, 0.68)	Ref	
	Up to 10	0.11 (–0.50, 0.71)	0.07 (0.05, 0.08)		0.14 (–0.47, 0.72)	0.04 (0.03, 0.06)	
	Between 11 and 20	0.02 (–0.60, 0.62)	–0.003 (–0.02, 0.01)		0.03 (–0.59, 0.63)	–0.05 (–0.07, –0.04)	
	Between 21 and 30	–0.07 (–0.69, 0.53)	–0.09 (–0.10, –0.07)		–0.10 (–0.72, 0.51)	–0.17 (–0.19, –0.16)	
	More than 30	–0.24 (–0.89, 0.40)	–0.22 (–0.24, –0.20)		–0.33 (–1.01, 0.31)	–0.39 (–0.41, –0.37)	
Yes	No	0.06 (–0.54, 0.66)	0.02 (0.005, 0.03)		0.11 (–0.49, 0.70)	0.01 (0.004, 0.02)	
	Up to 10	0.14 (–0.46, 0.74)	0.09 (0.06, 0.11)	0.01 (–0.03, 0.04)	0.16 (–0.44, 0.75)	0.06 (0.03, 0.08)	0.0005 (–0.03, 0.03)
	Between 11 and 20	0.02 (–0.59, 0.64)	–0.009 (–0.03, 0.01)	–0.02 (–0.05, 0.01)	0.01 (–0.61, 0.60)	–0.08 (–0.10, –0.06)	–0.04 (–0.07, –0.01)
	Between 21 and 30	–0.10 (–0.71, 0.50)	–0.12 (–0.15, –0.09)	–0.05 (–0.09, –0.01)	–0.16 (–0.79, 0.45)	–0.24 (–0.27, –0.21)	–0.08 (–0.12, –0.04)
	More than 30	–0.26 (–0.90, 0.36)	–0.26 (–0.29, –0.22)	–0.05 (–0.10, –0.003)	–0.38 (–1.07, 0.25)	–0.46 (–0.50, –0.43)	–0.09 (–0.13, –0.04)

The spirometry measurements are standardized by age, sex and height with a mean of 0 and a standard deviation of 1.

a Estimates obtained from a marginal structural model. The probability of maternal smoking is predicted based on participant’s age, sex, qualifications, income, Townsend area-level deprivation index and ethnicity. The probability of own smoking is predicted based on maternal smoking, in addition to the participant’s age, sex, qualifications, income, Townsend area-level deprivation index, ethnicity, asthma at baseline, height and BMI.

**Table 3.** Multiple-imputation analysis of maternal smoking in relation to FVC and FEV_1_ at baseline according to own smoking (*n* = 502 626)

Spirometry measurements	Pack-years of own smoking	Mean difference (95% CI)^a^
FVC	No	0.02 (0.005, 0.03)
	Up to 10	0.02 (–0.009, 0.05)
	Between 11 and 20	–0.006 (–0.03, 0.02)
	Between 21 and 30	–0.03 (–0.07, 0.005)
	More than 30	–0.03 (–0.08, 0.01)
FEV_1_	No	0.01 (0.004, 0.025)
	Up to 10	0.01 (–0.02, 0.05)
	Between 11 and 20	–0.03 (–0.05, 0.002)
	Between 21 and 30	–0.07 (–0.10, –0.03)
	More than 30	–0.07 (–0.12, –0.03)

The spirometry measurements are standardized by age, sex and height with a mean of 0 and a standard deviation of 1.

a Estimates of associations with maternal smoking within groups of pack-years of own smoking are obtained from a marginal structural model. The probability of maternal smoking is predicted based on participant’s age, sex, qualifications, income, Townsend area-level deprivation index and ethnicity. The probability of own smoking is predicted based on maternal smoking, in addition to the participant’s age, sex, qualifications, income, Townsend area-level deprivation index, ethnicity, asthma at baseline, height and BMI.

There was heterogeneity in the associations of maternal and own smoking with FEV_1_/FVC between the sexes (*p*-value < 0.001) and stratified results are therefore presented. In line with the findings for FEV_1_ and FVC, the reduction in FEV_1_/FVC among those exposed to both maternal and own smoking also exceeded what was expected based on the independent associations. For example, among women, the excess reduction in FEV_1_/FVC due to maternal smoking was –0.02, –0.01, –0.11 and –0.11 among those who had smoked up to 10, 11–20, 21–30 and more than 30 pack-years, respectively (Table 4). There was evidence of an inverse association with maternal smoking in the two highest categories of own smoking in both sexes, where the mean difference in FEV_1_/FVC was –0.11 (95% CI: –0.19, –0.04) among women who had smoked more than 30 years (Table 5). The results from the complete-case analysis showed a similar trend (Supplementary Tables 3 and 4, available as Supplementary data at *IJE* online). An excess reduction in FEV1/FVC due to the combined exposure of maternal and pack-years of own smoking was observed for both former and current smokers (Supplementary Table 5, available as Supplementary data at *IJE* online).

**Table 4.** Multiple-imputation analysis of maternal and pack-years of own smoking in relation to FEV_1_/FVC at baseline stratified by sex

Sex	Maternal smoking	Pack-years of own smoking	Median (IQR)	Mean difference (95% CI)^a^	Additive interaction
					Relative excess change (95% CI)
Women (*n* = 273 456)	No	No	0.19 (–0.41, 0.71)	Ref	
		Up to 10	0.13 (–0.46, 0.66)	–0.05 (–0.07, –0.03)	
		Between 11 and 20	0.04 (–0.59, 0.58)	–0.17 (–0.19, –0.15)	
		Between 21 and 30	–0.06 (–0.73, 0.50)	–0.28 (–0.31, –0.26)	
		More than 30	–0.28 (–1.04, 0.33)	–0.53 (–0.57, –0.51)	
	Yes	No	0.18 (–0.41, 0.69)	0.0002 (–0.01, 0.01)	
		Up to 10	0.11 (–0.48, 0.63)	–0.07 (–0.10, –0.03)	–0.02 (–0.06, 0.02)
		Between 11 and 20	0.02 (–0.62, 0.56)	–0.18 (–0.21, –0.15)	–0.01 (–0.05, 0.03)
		Between 21 and 30	–0.17 (–0.86, 0.42)	–0.39 (–0.44, –0.34)	–0.11 (–0.16, –0.05)
		More than 30	–0.39 (–1.18, 0.26)	–0.65 (–0.72, –0.58)	–0.11 (–0.19, –0.04)
Men (*n* = 229 170)	No	No	0.22 (–0.40, 0.73)	Ref	
		Up to 10	0.17 (–0.44, 0.69)	–0.04 (–0.06, –0.02)	
		Between 11 and 20	0.14 (–0.48, 0.66)	–0.07 (–0.10, –0.05)	
		Between 21 and 30	0.05 (–0.60, 0.59)	–0.17 (–0.19, –0.15)	
		More than 30	–0.18 (–0.91, 0.42)	–0.40 (–0.42, –0.38)	
	Yes	No	0.21 (–0.41, 0.72)	–0.001 (–0.02, 0.01)	
		Up to 10	0.16 (–0.45, 0.68)	–0.04 (–0.08, –0.004)	0.001 (–0.04, 0.04)
		Between 11 and 20	0.05 (–0.59, 0.59)	–0.15 (–0.19, –0.12)	–0.08 (–0.12, –0.03)
		Between 21 and 30	–0.04 (–0.71, 0.53)	–0.24 (–0.28, –0.20)	–0.07 (–0.11, –0.02)
		More than 30	–0.26 (–1.01, 0.37)	–0.47 (–0.51, –0.43)	–0.07 (–0.11, –0.02)

The spirometry measurements are standardized by age, sex and height with a mean of 0 and a standard deviation of 1.

a Estimates obtained from a marginal structural model. The probability of maternal smoking is predicted based on participant’s age, sex, qualifications, income, Townsend area-level deprivation index and ethnicity. The probability of own smoking is predicted based on maternal smoking, in addition to the participant’s age, sex, qualifications, income, Townsend area-level deprivation index, ethnicity, asthma at baseline, height and BMI.

**Table 5.** Multiple-imputation analysis of maternal smoking in relation to FEV_1_/FVC at baseline according to own smoking stratified by sex

Sex	Pack-years of own smoking	Mean difference (95% CI)
Women (*n* = 273 456)	No	0.0003 (–0.01, 0.01)
	Up to 10	–0.02 (–0.06, 0.02)
	Between 11 and 20	–0.01 (–0.05, 0.02)
	Between 21 and 30	–0.11 (–0.16, –0.05)
	More than 30	–0.11 (–0.19, –0.04)
Men (*n* = 229 170)	No	–0.001 (–0.02, 0.01)
	Up to 10	0.00005 (–0.04, 0.04)
	Between 11 and 20	–0.08 (–0.12, –0.04)
	Between 21 and 30	–0.07 (–0.11, –0.02)
	More than 30	–0.07 (–0.11, –0.03)

The spirometry measurements are standardized by age, sex and height with a mean of 0 and a standard deviation of 1.

Estimates of associations with maternal smoking within groups of pack-years of own smoking are obtained from a marginal structural model. The probability of maternal smoking is predicted based on participant’s age, sex, qualifications, income, Townsend area-level deprivation index and ethnicity. The probability of own smoking is predicted based on maternal smoking, in addition to the participant’s age, sex, qualifications, income, Townsend area-level deprivation index, ethnicity, asthma at baseline, height and BMI.

433 863 participants free of COPD at baseline were included in the analysis of incident hospitalization/death from COPD. We found no strong evidence of an association between maternal smoking and hospitalization/death from COPD among never smokers (Table 6). The combined exposure to maternal and own smoking was associated with a risk of hospitalization/death from COPD that exceed those exposed to only one or the other (Table 6). This excess risk due to maternal smoking was u-shaped, with values of 1.72, 0.30, 1.05 and 1.27 among those who smoked up to 10, 11–20, 21–30 and more than 30 pack-years, respectively (Table 6). In line with the interaction, the association with maternal smoking varied across categories of own smoking, with HRs of 2.25, 1.23, 1.30 and 1.14 among those who had smoked up to 10, 11–20, 21–30 and more than 30 pack-years, respectively (Table 7). The findings were similar in the complete-case analysis (Supplementary Tables 6 and 7, available as Supplementary data at *IJE* online). We also evaluated potential differences according to whether the participant was a former or current smoker (Supplementary Table 8, available as Supplementary data at *IJE* online). Among current smokers, the direction of the RERI was positive for the three lowest categories of pack-years of own smoking and negative for the highest category of own smoking. However, the CIs for the RERI all included the null value, likely reflecting the small number of exposed cases.

**Table 6.** Multiple-imputation analysis of maternal and pack-years of own smoking in relation to incident hospitalization/death from chronic obstructive pulmonary disease (*n* = 433 863)

Maternal smoking	Pack-years of own smoking	*N*	*n* (%) cases	HR (95% CI)^a^	Additive interaction
					Relative excess change (95% CI)
No	No	209, 546	247 (0.1)	1	
	Up to 10	29, 226	55 (0.2)	1.61 (1.15, 2.26)	
	Between 11 and 20	28, 384	115 (0.4)	2.70 (2.04, 3.57)	
	Between 21 and 30	18, 658	146 (0.8)	4.49 (3.46, 5.82)	
	More than 30	21, 373	514 (2.4)	11.33 (9.20, 13.97)	
Yes	No	79, 445	120 (0.2)	1.32 (0.98, 1.77)	
	Up to 10	11, 141	46 (0.4)	3.62 (2.32, 5.66)	1.72 (0.10, 3.34)
	Between 11 and 20	12, 789	68 (0.5)	3.32 (2.32, 4.76)	0.30 (–1.14, 1.75)
	Between 21 and 30	10, 040	94 (0.9)	5.85 (3.92, 8.74)	1.05 (–1.34, 3.44)
	More than 30	13, 261	375 (2.8)	12.94 (10.34, 16.19)	1.27 (–1.57, 4.12)

a Estimates obtained from a marginal structural model. The inverse probability of maternal smoking is predicted based on participant’s age, sex, qualifications, income, Townsend area-level deprivation index and ethnicity. The inverse probability of own smoking is predicted based on maternal smoking, in addition to the participant’s age, sex, qualifications, income, Townsend area-level deprivation index, ethnicity, asthma at baseline, height and BMI.

**Table 7.** Multiple-imputation analysis of maternal smoking in relation to incident hospitalization/death from chronic obstructive pulmonary disease according to own smoking (n = 433 863)

Pack-years of own smoking	HR (95% CI)
No	1.32 (0.98, 1.77)
Up to 10	2.25 (1.30, 3.89)
Between 11 and 20	1.23 (0.80, 1.89)
Between 21 and 30	1.30 (0.85, 2.01)
More than 30	1.14 (0.91, 1.43)

Estimates of associations with maternal smoking within groups of pack-years of own smoking are obtained from a marginal structural model.

The probability of maternal smoking is predicted based on participant’s age, sex, qualifications, income, Townsend area-level deprivation index and ethnicity. The probability of own smoking is predicted based on maternal smoking, in addition to the participant’s age, sex, qualifications, income, Townsend area-level deprivation index, ethnicity, asthma at baseline, height and BMI.

The combined exposure to maternal and own smoking was also associated with a risk of prevalent COPD at baseline that exceeds their individual associations (Supplementary Table 9, available as Supplementary data at *IJE* online). The excess risk due to maternal smoking was 0.04, 0.14, 0.13 and 0.33 among women who had smoked up to 10, 11–20, 21–30 and more than 30 pack-years, respectively (Supplementary Table 9, available as Supplementary data at *IJE* online). There was weak evidence for a positive association between maternal smoking and COPD at baseline in the two highest categories of own smoking (Supplementary Table 10, available as Supplementary data at *IJE* online). The results from the sensitivity analysis excluding individuals with asthma at baseline from the analysis of prevalent and incident COPD yielded similar results.

## Discussion

In this large-scale study, exposure to both maternal and own smoking was associated with an increased risk of COPD and reduction in lung function that exceeded what was expected based on their independent associations. Whereas the interaction between maternal and own smoking showed some evidence of a dose response for lung function and COPD at baseline, it was only observed in the lowest category of own smoking for incident hospitalization/death from COPD. The association with maternal smoking therefore varied across categories of pack-years of own smoking. There was no strong evidence that maternal smoking was associated with lung function or hospitalization/death from COPD among never smokers.

A limited number of previous studies examined maternal smoking in relation to adult lung function or COPD.^4^^,^^7–9^ A previous UK study of 2195 individuals between 30 and 59 years of age reported that maternal smoking before birth was associated with reduced lung volume irrespective of own smoking and appeared to interact with own smoking to increase airflow limitation and COPD risk.^7^ Another study of 18 922 subjects aged 20–44 years participating in the European Community Respiratory Health Survey reported that both maternal smoking during pregnancy and environmental tobacco smoke exposure during childhood were associated with more respiratory symptoms and poorer lung function in adulthood.^8^ Results from the same cohort also indicated an interaction between maternal and own smoking on the rate of lung-function decline.^9^ The previous studies that attempted to examine an interaction between maternal and own smoking used a rather crude categorization of own smoking (never, former and current). In the current study, we used information on pack-years of own smoking and were able to show the complex nuances in this interaction.

There are several potential non-exclusive explanatory mechanisms for an influence of maternal smoking on adult lung health. Since maternal smoking during pregnancy is associated with low birth weight and preterm delivery,^18^^,^^19^ which are linked to reduced lung function in adulthood,^20–22^ this underlying disadvantage in the lung development of offspring born to mothers who smoke during pregnancy might render them more vulnerable to COPD in adulthood. Changes in DNA methylation and telomere length shortening are other potential explanations for a direct effect of maternal smoking during pregnancy on lung function and hospitalization/death from COPD.^23^^,^^24^

Individuals exposed to maternal smoking might also be more susceptible to adverse effects of their own smoking on lung health. This interaction between maternal and own smoking in relation to hospitalization/death from COPD may be driven by an association between COPD risk genes and childhood lung response to maternal smoking.^25^ Furthermore, maternal smoking during pregnancy is associated with lower lung function during childhood, which subsequently tracks through adulthood,^5^ and COPD is associated with lower lung function at age 40 years.^26^ It is therefore plausible that the combination of a lower maximally attained lung function due to maternal smoking, together with a more rapid decline in lung function due to own smoking, results in a particularly elevated risk of COPD. These mechanisms would explain why we found the greatest excess reduction in FEV_1_/FVC and excess risk of prevalent COPD at baseline associated with maternal smoking in the highest category of own smoking. An explanation for our observation that the magnitude of the excess risk due to maternal smoking in relation to hospitalization/death from COPD was highest in the two extreme categories of own smoking could be that individuals exposed to both maternal and own smoking experience an earlier disease onset. If this is the case, the study population included in the evaluation of incident hospitalization/death from COPD might constitute healthier/more resilient people. However, further evidence is necessary to clarify whether this might be the case.

The main strengths of the current study is the size, which provided adequate power to test for an interaction between maternal smoking and different categories of pack-years of own smoking, the prospective follow-up and linkage to national registers. Our study also has limitations. The low participation rate in UK Biobank suggests potential for selection bias. The proportion of current smokers in UK Biobank was lower than estimates from the UK Office of National Statistics (11 vs 20%).^27^ This selection will influence the generalizability of our findings, but does not necessarily influence the internal validity.^28–30^ Due to the fact that the spirometry measurements were not conducted post bronchodilation, we might have over-estimated the number of individuals we classified with COPD at baseline. Our choice of using a fixed ratio of FEV_1_/FVC <0.70 as a proxy for prevalent COPD has limitations. Using this criterion might have contributed to an over-estimation of COPD due to the age of the UK Biobank participants. The alternative would have been to use cut-off levels based on the lower limit of normal for FEV_1_/FVC. However, this alternative approach has its own limitations, as it is dependent on the reference equations that you use, and it has not been validated in longitudinal studies.^12^ We also acknowledge that incident hospitalization/death from COPD, as captured from the hospital and death registers, reflects more severe COPD cases. Using the information available to us, we were not able to capture incident cases of COPD that occurred after baseline that did not require hospitalization and were not registered on the death certificate.

Asking adults to recall maternal smoking may also have resulted in misclassification. Whereas adults might recall maternal smoking during their childhood years, they cannot remember maternal smoking around the time of their delivery. We might therefore speculate that adult offspring are more likely to report that their mother smoked around the time of their delivery if she continued smoking during their childhood years. However, a validation study from the US Nurses Health Study indicated a reasonable validity of offspring’s report that their mother smoked around the time of their delivery compared with asking the mothers directly.^31^ Despite these findings, we cannot exclude an influence of differential misclassification of maternal and/or own smoking. For example, heavy smokers might be more likely both to under-report their own smoking and to recall that their mothers smoked. Such a differential misclassification might have lead us to underestimate the associations with own smoking and overestimate the association with maternal smoking. It is also possible that participants changed their smoking status after the baseline follow-up. A repeat assessment was conducted for 20 000 UK Biobank participants a median of 4 years after the main data collection. Of the participants who reported that they were current smokers at baseline, 36% reported that they had quit at the follow-up visit. For individuals who continued smoking after baseline, we likely underestimated their amount of exposure to own smoking, depending on how much they smoked after baseline and the timing of hospitalization/death from COPD. Lastly, since we did not have any direct measures of maternal socio-economic position, we cannot exclude unmeasured confounding.

In conclusion, our findings indicate an excess reduction of FEV_1_/FVC and risk of COPD due to maternal smoking that is heterogeneous across levels of own smoking. This further emphasizes the importance of smoking avoidance and cessation for lung health, and suggests that public-health initiatives supporting the current trend of reduction in smoking may have benefits for two generations.

## Funding

This work was supported by the UK Medical Research Council (grant number MC_UU_12013/5, MR/M009351/1 to A.F. and MR/M020894/1 to L.D.H.). This study was also supported by the National Institute for Health Research Biomedical Research Centre at the University Hospitals Bristol National Health Service Foundation Trust and the University of Bristol. This work was also partly supported by the Research Council of Norway through its Centres of Excellence funding scheme, project number 262700. The views expressed in this publication are those of the author(s) and not necessarily the funders.

## Supplementary Material

Supplementary Figure and Tables
