# Supplementary material for: Independent and combined associations of maternal and own smoking with adult lung function and COPD
Source: Int J Epidemiol. 2018 Oct 17;47(6):1855–64. doi: 10.1093/ije/dyy221 (PMC6280943; doi:10.1093/ije/dyy221)
Supplement: Supplementary Figure and Tables [file dyy221_supplementary_figure-tables.docx]

Supplementary Figure 1 Illustration of sample size available for the complete-case analysis

Information on potential confounders (n= 262,239)

Number of participants in UK Biobank (n=502,626)

Information on maternal smoking (n= 367,060)

Lung function measurements (n= 266,693)

Information on pack years of own smoking (n=423,713)

Supplementary Table 1 Complete-case analysis of maternal and pack years of own smoking in relation to FVC and FEV_1_ at baseline

| Maternal smoking | Pack years of own smoking | FVC | | | FEV_1_ | | |
| --- | --- | --- | --- | --- | --- | --- | --- |
|  |  | Median (IQR) | Mean difference  (95% CI) ^a^ | Additive interaction  Relative excess change (95% CI) | Median (IQR) | Mean difference (95% CI) ^a^ | Additive interaction  Relative excess change (95% CI) |
| No | No | 0.02 (-0.61, 0.65) | Ref |  | 0.09 (-0.54, 0.69) | Ref |  |
|  | Up to 10 | 0.13 (-0.47, 0.72) | 0.10 (0.09, 0.12) |  | 0.17 (-0.44, 0.74) | 0.07 (0.06, 0.09) |  |
|  | Between 11 and 20 | 0.02 (-0.60, 0.61) | -0.01 (-0.03, 0.01) |  | 0.03 (-0.58, 0.63) | -0.06 ( -0.08, -0.04) |  |
|  | Between 21 and 30 | -0.10 (-0.71, 0.49) | -0.13 (-0.15, -0.11) |  | -0.13 (-0.75, 0.46) | -0.23 (-0.25, -0.21) |  |
|  | More than 30 | -0.34 (-0.97, 0.31) | -0.35 (-0.36, -0.33) |  | -0.45 (-1.13, 0.18) | -0.56 (-0.58, -0.54) |  |
| Yes | No | 0.07 (-0.52, 0.67) | 0.04 (0.03, 0.05) |  | 0.14 (-0.45, 0.71) | 0.04 (0.02, 0.05) |  |
|  | Up to 10 | 0.18 (-0.41, 0.77) | 0.15 (0.12, 0.17) | 0.01 (-0.02, 0.04) | 0.20 (-0.38, 0.78) | 0.11 (0.08, 0.13) | 0.001 (-0.03, 0.03) |
|  | Between 11 and 20 | 0.03 (-0.57, 0.64) | 0.002 (-0.02, 0.03) | -0.03 (-0.06, 0.01) | 0.03 (-0.60, 0.60) | -0.08 (-0.10, -0.05) | -0.05 (-0.08, -0.02) |
|  | Between 21 and 30 | -0.13 (-0.73, 0.45) | -0.16 (-0.18, -0.13) | -0.06 (-0.09, -0.03) | -0.19 (-0.82, 0.40) | -0.29 (-0.32, -0.27) | -0.10 (-0.13, -0.06) |
|  | More than 30 | -0.34 (-0.97, 0.26) | -0.37 (-0.39, -0.35) | -0.06 (-0.09, -0.03) | -0.50 (-1.19, 0.13) | -0.61 (-0.64, -0.59) | -0.09 (-0.12, -0.06) |

(n=262,239)

The spirometry measurements are standardized by age, sex and height with a mean of zero and a standard deviation of one.

^a^ Estimates obtained from a marginal structural model. The probability of maternal smoking is predicted based on participant’s age, sex, qualifications, income, Townsend area-level deprivation index and ethnicity. The probability of own smoking is predicted based on maternal smoking, in addition to the participant’s age, sex, qualifications, income, Townsend area-level deprivation index, ethnicity, asthma at baseline, height and BMI.

Supplementary Table 2 Complete-case analysis of maternal smoking in relation to FVC and FEV_1_ at baseline according to own smoking

(n=262,239)

| Spirometry measurements | Pack years of own smoking | Mean difference  (95 % CI) |
| --- | --- | --- |
| FVC | No | 0.03 (0.02, 0.04) |
|  | Up to 10 | 0.04 (0.01, 0.07) |
|  | Between 11 and 20 | 0.01 (-0.02, 0.03) |
|  | Between 21 and 30 | -0.02 (-0.05, 0.01) |
|  | More than 30 | -0.03 (-0.05, 0.001) |
| FEV_1_ | No | 0.04 (0.02, 0.05) |
|  | Up to 10 | 0.04 (0.01, 0.07) |
|  | Between 11 and 20 | -0.02 (-0.04, 0.01) |
|  | Between 21 and 30 | -0.06 (-0.09, -0.03) |
|  | More than 30 | -0.05 (-0.08, -0.02) |

The spirometry measurements are standardized by age, sex and height with a mean of zero and a standard deviation of one.

Estimates of associations with maternal smoking within groups of pack years of own smoking are obtained from a marginal structural model. The probability of maternal smoking is predicted based on participant’s age, sex, qualifications, income, Townsend area-level deprivation index and ethnicity. The probability of own smoking is predicted based on maternal smoking, in addition to the participant’s age, sex, qualifications, income, Townsend area-level deprivation index, ethnicity, asthma at baseline, height and BMI.

Supplementary Table 3 Complete-case analysis of maternal and pack years of own smoking in relation to FEV_1_/FVC stratified by sex

| Sex | Maternal smoking | Pack years of own smoking | Median (IQR) | Mean difference  (95% CI) ^a^ | Additive interaction  Relative excess change  (95% CI) |
| --- | --- | --- | --- | --- | --- |
| Women  (n= 143,885) | No | No | 0.23 (-0.34, 0.71) | Ref |  |
|  |  | Up to 10 | 0.16 (-0.40, 0.65) | -0.05 (-0.07, -0.03) |  |
|  |  | Between 11 and 20 | 0.07 (-0.56, 0.58) | -0.17 (-0.20, -0.15) |  |
|  |  | Between 21 and 30 | -0.07 (-0.73, 0.47) | -0.33 (-0.35, -0.30) |  |
|  |  | More than 30 | -0.37 (-1.18, 0.25) | -0.71 (-0.74, -0.67) |  |
|  | Yes | No | 0.22 (-0.34, 0.70) | 0.004 (-0.01, 0.02) |  |
|  |  | Up to 10 | 0.14 (-0.43, 0.61) | -0.08 (-0.11, -0.04) | -0.03 (-0.07, 0.01) |
|  |  | Between 11 and 20 | 0.03 (-0.58, 0.55) | -0.19 (-0.23, -0.16) | -0.02 (-0.07, 0.02) |
|  |  | Between 21 and 30 | -0.19 (-0.89, 0.38) | -0.45 (-0.49, -0.40) | -0.12 (-0.18, -0.07) |
|  |  | More than 30 | -0.49 (-1.39, 0.17) | -0.81 (-0.86, -0.77) | -0.11 (-0.17, -0.05) |
| Men  (n=118,354) | No | No | 0.25 (-0.34, 0.74) | Ref |  |
|  |  | Up to 10 | 0.20 (-0.39, 0.69) | -0.05 (-0.07, -0.02) |  |
|  |  | Between 11 and 20 | 0.18 (-0.43, 0.67) | -0.08 (-0.10, -0.05) |  |
|  |  | Between 21 and 30 | 0.06 (-0.60, 0.58) | -0.22 (-0.25, -0.19) |  |
|  |  | More than 30 | -0.23 (-1.00, 0.37) | -0.55 (-0.57, -0.52) |  |
|  | Yes | No | 0.24 (-0.35, 0.72) | 0.001 (-0.01, 0.02) |  |
|  |  | Up to 10 | 0.21 (-0.37, 0.68) | -0.03 (-0.06, 0.01) | 0.02 (-0.03, 0.06) |
|  |  | Between 11 and 20 | 0.07 (-0.57, 0.57) | -0.16 (-0.20, -0.13) | -0.09 (-0.13, -0.05) |
|  |  | Between 21 and 30 | -0.05 (-0.71, 0.51) | -0.29 (-0.33, -0.25) | -0.07 (-0.12, -0.02) |
|  |  | More than 30 | -0.31 (-1.10, 0.34) | -0.59 (-0.63, -0.56) | -0.05 (-0.09, -0.01) |

The spirometry measurements are standardized by age, sex and height with a mean of zero and a standard deviation of one.

^a^ Estimates obtained from a marginal structural model. The probability of maternal smoking is predicted based on participant’s age, sex, qualifications, income, Townsend area-level deprivation index and ethnicity. The probability of own smoking is predicted based on maternal smoking, in addition to the participant’s age, sex, qualifications, income, Townsend area-level deprivation index, ethnicity, asthma at baseline, height and BMI.

Supplementary Table 4 Complete-case analysis of maternal smoking in relation to FEV_1_/FVC according to own smoking stratified by sex

| Sex | Pack years of own smoking | Mean difference (95 % CI) |
| --- | --- | --- |
| Women  (n=143,875) | No | 0.004 ( -0.01, 0.02) |
|  | Up to 10 | -0.02 ( -0.06, 0.01) |
|  | Between 11 and 20 | -0.01 (-0.06, 0.02) |
|  | Between 21 and 30 | -0.12 (-0.17, -0.07) |
|  | More than 30 | -0.11 (-0.16, -0.05) |
| Men  (n=118,344) | No | 0.001 (-0.01, 0.02) |
|  | Up to 10 | 0.02 (-0.02, 0.06) |
|  | Between 11 and 20 | -0.09 (-0.13, -0.05) |
|  | Between 21 and 30 | -0.07 (-0.12, -0.02) |
|  | More than 30 | -0.05 (-0.09, -0.01) |

The spirometry measurements are standardized by age, sex and height with a mean of zero and a standard deviation of one.

Estimates of associations with maternal smoking within groups of pack years of own smoking are obtained from a marginal structural model. The probability of maternal smoking is predicted based on participant’s age, sex, qualifications, income, Townsend area-level deprivation index and ethnicity. The probability of own smoking is predicted based on maternal smoking, in addition to the participant’s age, sex, qualifications, income, Townsend area-level deprivation index, ethnicity, asthma at baseline, height and BMI.

Supplementary Table 5 Multiple imputation analysis examining differences among former and current smokers in associations of maternal and pack years of own smoking in relation to FEV_1_/FVC

| Maternal smoking | Smoking history | Pack years of own smoking | Women (n=273,456) | | Men (n= 229,170) | |
| --- | --- | --- | --- | --- | --- | --- |
|  |  |  | Mean difference (95% CI) ^a^ | Additive interaction  Relative excess change (95% CI) | Mean difference (95% CI) ^a^ | Additive interaction  Relative excess change (95% CI) |
| No | Never | 0 No | Ref |  | Ref |  |
|  | Former | 1 Up to 10 | -0.015 (-0.035, 0.005) |  | -0.004 (-0.026, 0.017) |  |
|  |  | 2 Between 11 and 20 | -0.108 (-0.131, 0.085) |  | -0.019 (-0.043, 0.006) |  |
|  |  | 3 Between 21 and 30 | -0.192 (-0.223, -0.161) |  | -0.089 (-0.115, -0.063) |  |
|  |  | 4 More than 30 | -0.416 (-0.455, -0.376) |  | -0.299 (-0.325, -0.273) |  |
|  | Current | 5 Up to 10 | -0.233 (-0.280, -0.187) |  | -0.207 (-0.253, -0.162) |  |
|  |  | 6 Between 11 and 20 | -0.366 (-0.411, -0.320 |  | -0.284 (-0.327, -0.241) |  |
|  |  | 7 Between 21 and 30 | -0.479 (-0.525, -0.433) |  | -0.395 (-0.438, -0.353) |  |
|  |  | 8 More than 30 | -0.715 (-0.768, -0.661) |  | -0.598 (-0.639, -0.557) |  |
| Yes | Never | 9 No | 0.0002 (-0.012, 0.012) |  | -0.0009 (-0.016, 0.015) |  |
|  | Former | 10 Up to 10 | -0.037 (-0.071, -0.003) | -0.022 (-0.061, 0.017) | -0.003 (-0.039, 0.032) | 0.002 (-0.040, 0.044) |
|  |  | 11 Between 11 and 20 | -0.103 (-0.136, -0.070) | 0.005 (-0.035, 0.045) | -0.088 (-0.121, -0.054) | -0.068 (-0.112, -0.024) |
|  |  | 12 Between 21 and 30 | -0.298 (-0.360, -0.235) | -0.105 (-0.176, -0.035) | -0.150 (-0.195, -0.105) | -0.060 (-0.114, -0.007) |
|  |  | 13 More than 30 | -0.554 (-0.644, -0.463) | -0.138 (-0.241, -0.035) | -0.346 (-0.392, -0.299) | -0.046 (-0.097, 0.005) |
|  | Current | 14 Up to 10 | -0.256 (-0.357, -0.155) | -0.023 (-0.138, 0.093) | -0.212 (-0.307, -0.117) | -0.005 (-0.112, 0.103) |
|  |  | 15 Between 11 and 20 | -0.437 (-0.514, -0.360) | -0.072 (-0.163, 0.020) | -0.378 (-0.470, -0.285) | -0.093 (-0.196, 0.010) |
|  |  | 16 Between 21 and 30 | -0.582 (-0.665, -0.500) | -0.103 (-0.196, -0.010) | -0.457 (-0.536, -0.379) | -0.061 (-0.150, 0.027) |
|  |  | 17 More than 30 | -0.792 (-0.905, -0.680) | -0.080 (-0.205, 0.049) | -0.689 (-0.758, -0.620) | -0.090 (-0.174, -0.007) |

(n=502,626)

The spirometry measurements are standardized by age, sex and height with a mean of zero and a standard deviation of one.

^a^ Estimates obtained from a marginal structural model. The probability of maternal smoking is predicted based on participant’s age, sex, qualifications, income, Townsend area-level deprivation index and ethnicity. The probability of own smoking is predicted based on maternal smoking, in addition to the participant’s age, sex, qualifications, income, Townsend area-level deprivation index, ethnicity, history of asthma at recruitment, height and BMI.

Supplementary Table 6 Complete-case analysis of maternal and pack years of own smoking in relation to incident hospitalization/death from chronic obstructive pulmonary disease

(n=230,840)

| Maternal smoking | Pack years of own smoking | N | n (%) cases | HR (95% CI) ^a^ | Additive interaction  Relative excess risk (95% CI) |
| --- | --- | --- | --- | --- | --- |
| No | No | 110,921 | 107 (0.10) | 1 |  |
|  | Up to 10 | 15,690 | 20 (0.13) | 1.46 (0.90, 2.38) |  |
|  | Between 11 and 20 | 14,680 | 35 (0.24) | 2.55 (1.73, 3.77) |  |
|  | Between 21 and 30 | 9,333 | 51 (0.55) | 6.01 (4.28, 8.45) |  |
|  | More than 30 | 9,742 | 190 (1.95) | 19.71 (15.50, 25.06) |  |
| Yes | No | 45,249 | 45 (0.10) | 0.98 (0.69, 1.40) |  |
|  | Up to 10 | 6,291 | 17 (0.27) | 2.66 (1.58, 4.49) | 1.22 (-0.26, 2.70) |
|  | Between 11 and 20 | 7,067 | 26 (0.37) | 4.02 (2.25, 7.18) | 1.49 (-0.90, 3.87) |
|  | Between 21 and 30 | 5,369 | 29 (0.54) | 4.72 (3.09, 7.22) | -1.26 (-3.78, 1.24) |
|  | More than 30 | 6,513 | 130 (2.00) | 18.13 (13.95, 23.57) | -1.55 (-5.91, 2.80) |

^a^ Estimates obtained from a marginal structural model. The inverse probability of maternal smoking is predicted based on participant’s age, sex, qualifications, income, Townsend area-level deprivation index and ethnicity. The inverse probability of own smoking is predicted based on maternal smoking, in addition to the participant’s age, sex, qualifications, income, Townsend area-level deprivation index, ethnicity, asthma at baseline, height and BMI.

Supplementary Table 7 Complete-case analysis of maternal smoking in relation to incident hospitalization/death from chronic obstructive pulmonary disease according to own smoking

(n=230,840)

| Pack years of own smoking | HR (95 % CI) |
| --- | --- |
| No | 0.98 (0.69, 1.40) |
| Up to 10 | 1.82 (0.94, 3.54) |
| Between 11 and 20 | 1.57 (0.83, 2.30) |
| Between 21 and 30 | 0.79 (0.49, 1.26) |
| More than 30 | 0.92 (0.73, 1.16) |

Estimates of associations with maternal smoking within groups of pack years of own smoking are obtained from a marginal structural model. The probability of maternal smoking is predicted based on participant’s age, sex, qualifications, income, Townsend area-level deprivation index and ethnicity. The probability of own smoking is predicted based on maternal smoking, in addition to the participant’s age, sex, qualifications, income, Townsend area-level deprivation index, ethnicity, asthma at baseline, height and BMI.

Supplementary Table 8 Multiple imputation analysis evaluating differences among former and current smokers in associations of maternal and pack years of own smoking with incident hospitalization/death from chronic obstructive pulmonary disease

| Maternal smoking | Smoking history | Pack years of own smoking | N | n (%) cases | HR (95% CI) ^a^ | Additive interaction  Relative excess change (95% CI) |
| --- | --- | --- | --- | --- | --- | --- |
| No | Never | 0 No | 208,426 | 246 (0.1) | Ref |  |
|  | Former | 1 Up to 10 | 24,471 | 42 (0.2) | 1.46 (1.00, 2.12) |  |
|  |  | 2 Between 11 and 20 | 22,275 | 68 (0.3) | 2.05 (1.48, 2.84) |  |
|  |  | 3 Between 21 and 30 | 13,475 | 92 (0.7) | 3.97 (2.93, 5.39) |  |
|  |  | 4 More than 30 | 14,610 | 282 (1.9) | 8.67 (6.92, 10.86) |  |
|  | Current | 5 Up to 10 | 4,605 | 13 (0.3) | 2.42 (1.28, 4.59) |  |
|  |  | 6 Between 11 and 20 | 5,936 | 46 (0.8) | 5.12 (3.44, 7.63) |  |
|  |  | 7 Between 21 and 30 | 5,060 | 53 (1.1) | 5.84 (4.14, 8.24) |  |
|  |  | 8 More than 30 | 6,573 | 228 (3.5) | 16.35 (12.45, 21.46) |  |
| Yes | Never | 9 No | 79,070 | 118 (0.1) | 1.31 (0.97, 1.76) |  |
|  | Former | 10 Up to 10 | 9,376 | 30 (0.3) | 2.65 (1.67, 4.20) | 0.89 (-0.40, 2.18) |
|  |  | 11 Between 11 and 20 | 9,877 | 36 (0.4) | 2.44 (1.51, 3.93) | 0.07 (-1.33, 1.48) |
|  |  | 12 Between 21 and 30 | 6,982 | 44 (0.6) | 4.61 (2.52, 8.45) | 0.33 (-2.65, 3.31) |
|  |  | 13 More than 30 | 8,598 | 211 (2.5) | 11.64 (8.86, 15.28) | 2.65 (-0.53, 5.84) |
|  | Current | 14 Up to 10 | 1,718 | 15 (0.9) | 8.98 (4.24, 18.98) | 6.37 (-0.51, 13.23) |
|  |  | 15 Between 11 and 20 | 2,842 | 32 (1.1) | 6.51 (4.04, 10.49) | 1.09 (-2.77, 4.95) |
|  |  | 16 Between 21 and 30 | 2,994 | 48 (1.6) | 8.69 (5.78, 13.06) | 2.56 (-1.17, 6.29) |
|  |  | 17 More than 30 | 4,552 | 160 (3.5) | 15.02 (11.14, 20.26) | -1.65 (-7.17, 3.87) |

(n=433,863)

^a^ Estimates obtained from a marginal structural model. The probability of maternal smoking is predicted based on participant’s age, sex, qualifications, income, Townsend area-level deprivation index and ethnicity. The probability of own smoking is predicted based on maternal smoking, in addition to the participant’s age, sex, qualifications, income, Townsend area-level deprivation index, ethnicity, asthma at baseline, height and BMI.

Supplementary Table 9 Multiple-imputation analysis of maternal and pack years of own smoking with prevalent chronic obstructive pulmonary disease at baseline

(n=502,626)

| Sex | Maternal smoking | Pack years of own smoking | N | n (%) cases | OR (95% CI) ^a^ | Additive interaction  Relative excess risk (95% CI) |
| --- | --- | --- | --- | --- | --- | --- |
| Women  (n= 273,456) | No | No | 139,356 | 12,878 (9.2) | Ref |  |
|  |  | Up to 10 | 18,088 | 1,769 (9.8) | 1.04 (0.97, 1.12) |  |
|  |  | Between 11 and 20 | 16,079 | 2,130 (13.3) | 1.43 (1.34, 1.52) |  |
|  |  | Between 21 and 30 | 10,528 | 1,803 (17.1) | 1.89 (1.76, 2.03) |  |
|  |  | More than 30 | 10,954 | 2,947 (26.9) | 3.07 (2.87, 3.30) |  |
|  | Yes | No | 49,539 | 4,291 (8.7) | 0.95 (0.91, 0.99) |  |
|  |  | Up to 10 | 7,287 | 707 (9.7) | 1.06 (0.94, 1.19) | 0.07 (-0.08, 0.22) |
|  |  | Between 11 and 20 | 7,835 | 1,006 (12.8) | 1.37 (1.25, 1.50) | -0.01 (-0.16, 0.14) |
|  |  | Between 21 and 30 | 6,130 | 1,093 (17.8) | 2.13 (1.90, 2.39) | 0.30 (0.03, 0.57) |
|  |  | More than 30 | 7,659 | 2,229 (29.1) | 3.46 (3.06, 3.92) | 0.44 (-0.03, 0.91) |
| Men  (n= 229,170) | No | No | 83,067 | 12,106 (12.7) | Ref |  |
|  |  | Up to 10 | 12,907 | 2,103 (14.0) | 1.10 (1.03, 1.17) |  |
|  |  | Between 11 and 20 | 14,436 | 2,887 (16.7) | 1.29 (1.20, 1.38) |  |
|  |  | Between 21 and 30 | 9,933 | 2,563 (20.5) | 1.60 (1.50, 1.70) |  |
|  |  | More than 30 | 13,366 | 6,157 (31.5) | 2.54 (2.43, 2.66) |  |
|  | Yes | No | 34,197 | 4,781 (12.3) | 0.97 (0.93, 1.02) |  |
|  |  | Up to 10 | 4,561 | 752 (14.2) | 1.11 (1.00, 1.25) | 0.04 (-0.10, 0.19) |
|  |  | Between 11 and 20 | 5,960 | 1,290 (17.8) | 1.41 (1.28, 1.55) | 0.14 (-0.02, 0.30) |
|  |  | Between 21 and 30 | 5,002 | 1,351 (21.3) | 1.70 (1.55, 1.87) | 0.13 (-0.05, 0.31) |
|  |  | More than 30 | 7,831 | 3921 (33.4) | 2.85 (2.64, 3.08) | 0.33 (0.09, 0.59) |

^a^ Estimates obtained from a marginal structural model. The probability of maternal smoking is predicted based on participant’s age, sex, qualifications, income, Townsend area-level deprivation index and ethnicity. The probability of own smoking is predicted based on maternal smoking, in addition to the participant’s age, sex, qualifications, income, Townsend area-level deprivation index, ethnicity, history of asthma at recruitment, height and BMI.

Supplementary Table 10 Multiple-imputation analysis of maternal smoking in relation to prevalent chronic obstructive pulmonary disease at baseline according to own smoking

(n=502,626)

| Sex | Pack years of own smoking | OR (95 % CI) |
| --- | --- | --- |
| Women (n= 273,456) | No | 0.95 (0.91, 0.99) |
|  | Up to 10 | 1.02 (0.89, 1.16) |
|  | Between 11 and 20 | 0.96 (0.86, 1.06) |
|  | Between 21 and 30 | 1.13 (0.99, 1.29) |
|  | More than 30 | 1.13 (0.98, 1.29) |
| Men (n= 229,170) | No | 0.97 (0.93, 1.02) |
|  | Up to 10 | 1.01 (0.89, 1.15) |
|  | Between 11 and 20 | 1.09 (0.97, 1.22) |
|  | Between 21 and 30 | 1.07 (0.96, 1.18) |
|  | More than 30 | 1.12 (1.03, 1.22) |

Estimates of associations with maternal smoking within groups of pack years of own smoking are obtained from a marginal structural model. The probability of maternal smoking is predicted based on participant’s age, sex, qualifications, income, Townsend area-level deprivation index and ethnicity. The probability of own smoking is predicted based on maternal smoking, in addition to the participant’s age, sex, qualifications, income, Townsend area-level deprivation index, ethnicity, history of asthma at recruitment, height and BMI.
